# Low-dose Initiation of Buprenorphine/naloxone for the Management of Chronic Non-cancer Pain in Patients on Long-term Opioid Therapy: A Case Series

**DOI:** 10.1080/24740527.2024.2310811

**Published:** 2024-01-26

**Authors:** Maya Rattanavong, Donica Kwan, Derek Jorgenson, Eric Landry, Radhika Marwah, Katelyn Halpape

**Affiliations:** a College of Pharmacy and Nutrition, University of Saskatchewan, Saskatoon, Saskatchewan, Canada; b Family Medicine, University of Saskatchewan, Saskatoon, Saskatchewan, Canada

**Keywords:** Buprenorphine/naloxone, microdosing, micro-induction, low-dose induction, low-dose initiation, Bernese method, chronic pain, opioid

## Abstract

**Background:**

Buprenorphine may provide superior analgesia to full opioid agonist therapy and reverse the effects of opioid-induced hyperalgesia, while having a favorable safety profile and fewer adverse effects, in chronic non-cancer pain treatment. Low-dose initiation of buprenorphine is a useful strategy for patients on long-term opioid therapy because it avoids the need for moderate opioid withdrawal required for traditional buprenorphine initiations. However, there are few published reports of low-dose initiation regimens in the setting of chronic pain.

**Aims:**

The aim of the study was to describe a case series of individuals living with chronic pain who were transitioned from long-term full opioid agonist therapy onto sublingual buprenorphine/naloxone using low-dose initiation regimens.

**Methods:**

This study is a retrospective case series that included all patients who received care at an outpatient chronic pain clinic and were scheduled for low-dose initiation of buprenorphine/naloxone between March 2020 and December 2022. Data were collected through a retrospective review of electronic medical records and results were analyzed using descriptive statistics.

**Results:**

Eighteen patients underwent transitions from their baseline opioids onto buprenorphine/naloxone using a low-dose initiation regimen. Of those patients, 17 successfully completed the initiation (94.44%), 12 experienced adverse effects during the initiation (66.67%), with only one patient requiring treatment discontinuation, and all adverse effects resolved once maintenance doses of buprenorphine/naloxone were established. The mean Clinical Global Impression–Improvement score after initiation was 2 (1–5).

**Conclusion:**

Low-dose initiation is an effective approach to transition patients with chronic non-cancer pain from long-term opioid therapy to buprenorphine/naloxone without major complications or worsening pain.

## RÉSUMÉ

**Contexte:** La buprénorphine peut offrir une analgésie supérieure à celle d’un traitement par agonistes opioïdes complet et inverser les effets de l’hyperalgésie induite par les opioïdes, tout en présentant un profil d’innocuité favorable et moins d’effets indésirables dans le traitement de la douleur chronique non cancéreuse. L’initiation à faible dose de la buprénorphine est une stratégie utile pour les patients sous traitement opioïde à long terme, car elle évite le besoin de sevrage des opioïdes modéré nécessaire pour les traitements traditionnels à base de buprénorphine. Cependant, il existe peu de rapports publiés sur les régimes d’initiation à faible dose dans le cadre de la douleur chronique.

**Objectifs:** L’objectif de cette étude était de décrire une série de cas d’individus vivant avec une douleur chronique qui sont passés d’un traitement opioïde complet à long terme à un traitement par buprénorphine sublinguale/naloxone en ayant recours à des régimes d’initiation à faible dose.

**Méthodes:** Cette étude est une série de cas rétrospective incluant tous les patients pris en charge dans une clinique externe de traitement de la douleur chronique et pour lesquels un schéma d’initiation à faible dose de buprénorphine/naloxone a été programmé entre mars 2020 et décembre 2022. Les données ont été collectées par le biais d’un examen rétrospectif des dossiers médicaux électroniques et les résultats ont été analysés à l’aide de statistiques descriptives.

**Résultats:** Dix-huit patients ont fait la transition des opioïdes de base à la buprénorphine/naloxone en utilisant un régime d’initiation à faible dose. Parmi ces patients, 17 ont terminé l’initiation avec succès (94,44 %), 12 ont présenté des effets indésirables pendant l’initiation (66,67 %) et un seul patient a dû interrompre son traitement. Tous les effets indésirables ont disparu une fois les doses d’entretien de buprénorphine/naloxone établies. Le score d’impression clinique globale-amélioration moyen après le début du traitement était de 2 (1-5).

**Conclusion:** L’initiation à faible dose est une approche efficace pour faire passer les patients souffrant de douleur chronique non cancéreuse d’un traitement opioïde à long terme à la buprénorphine/naloxone sans complications majeures ni aggravation de la douleur.

## Introduction

Buprenorphine is a semisynthetic opioid first developed as an analgesic in 1966.^1^ Due to its unique pharmacokinetics, mechanism of action, evidence for effectiveness, and safety profile, buprenorphine/naloxone (bup/nal) is a first-line treatment option for opioid use disorder (OUD).^2^ Fewer studies have been conducted evaluating the potential use of bup/nal in the setting of pain, particularly chronic non-cancer pain (CNCP), which is defined as pain persisting longer than 3 months.^3–5^ CNCP affects approximately 7.6 million Canadians, and this estimate is expected to rise in light of the growing proportion of older adults.^5^ Historically, prescription opioids were falsely promoted as low-risk, nonaddictive, and effective treatments for CNCP.^6^ However, this has long been disproven, as long-term opioid therapy (LTOT) can lead to many adverse effects, including opioid dependence and opioid tolerance. This may result in escalating opioid doses in an attempt to address uncontrolled pain.^6^ The relationship between chronic pain and LTOT has posed many challenges for health care providers and has given rise to the off-label use of bup/nal as a safer alternative for the management of CNCP, especially in the setting of comorbid opioid dependence, which is the physiologic adaption to chronic opioid use through the development of tolerance and withdrawal.^7,8^

Buprenorphine has a unique pharmacokinetic profile in that it is a partial mu‐opioid receptor (MOR) agonist, kappa‐opioid receptor antagonist, delta‐opioid receptor antagonist, and orphan‐like receptor 1 agonist.^7^ Compared to full opioid agonists, the action of buprenorphine at MORs produces a high-potency analgesic effect with fewer adverse effects, such as physical dependence, respiratory depression, and constipation.^7,9^ However, due to buprenorphine’s high MOR affinity and slow dissociation, the competitive binding of MORs can precipitate opioid withdrawal in the presence of full opioid agonists.^7,10^ As such, current bup/nal product monographs require patients to discontinue their existing opioid therapy and experience mild-to-moderate withdrawal symptoms prior to bup/nal initiation to avoid abrupt displacement at MORs.^11,12^ This practice is often referred to as “traditional initiation” and is the dosing strategy recommend in the bup/nal product monograph for OUD management.^11,12^

Opioid withdrawal symptoms, such as anxiety, sweating, and bone or joint pain, can cause significant patient discomfort, and avoiding withdrawal has been reported as the most important reason for current opioid use by patients with concurrent chronic pain and opioid dependence.^13,14^ Perceived intensity and emotional fear of withdrawal-associated pain can cause severe emotional and physical stress for certain patients and may be a significant barrier to successful opioid cessation.^15^ Opioid tapers may be an option for individuals living with CNCP who have been receiving LTOT with little or no benefit and/or are experiencing adverse effects.^16^ However, this approach increases the risk of accidental overdoses, opioid withdrawal, and pain crisis and has been associated with an increase in emergency department visits and hospitalizations.^17,18^

A more recent strategy termed “low-dose initiation,” also known as “microdosing” or the “Bernese method,” has emerged as an alternate buprenorphine initiation strategy that is hoped to eliminate the need for attaining withdrawal symptoms prior to traditional bup/nal initiation and also decrease the chance for precipitated withdrawals with buprenorphine.^18^ Low-dose initiation involves starting bup/nal at very low doses while the patient continues their existing opioid to allow for slow accumulation at opioid receptors, resulting in the gradual displacement of their full opioid agonist. This novel approach has grown increasingly common among prescribers for the management of OUD; however, there are sparse published data regarding its use in the setting of chronic pain.^7,19,20^

Published studies on low-dose initiation methods of buprenorphine have primarily focused on the treatment of OUD. There are nine published case reports/series describing the use of low-dose initiation bup/nal for the indication of CNCP in a total of 23 adult patients (Appendix). Of these 23 cases, 4 were initiated using transdermal buprenorphine before transitioning onto bup/nal.^19,21^ The remaining 20 cases were initiated using sublingual bup/nal with variable doses and durations of the initiation regimens.^8,22–27^ These cases highlight the potentially utility of low-dose bup/nal initiations in the treatment of patients with CNCP; however, there is a need for further research in this area to strengthen the evidence base for this practice.

This case series aims to summarize the use of low-dose initiation regimens of sublingual bup/nal in patients living with CNCP who were switched to sublingual bup/nal using low-dose initiation regimens at an interdisciplinary chronic pain clinic in Canada.

## Methods

This study is a case series that included patients who received chronic pain care at the USask Chronic Pain Clinic (UCPC) in Saskatoon, Saskatchewan, Canada, from March 2020 to December 2022. The information collected for this case series was gathered by retrospective review of electronic medical records. Results were analyzed using descriptive statistics. The protocol was approved by the University of Saskatchewan Research Ethics Board (Beh ID 2076), and all patients provided informed consent to research.

The UCPC provides interdisciplinary chronic pain care to patients alongside their current primary care team. The details of the UCPC are described elsewhere.^28,29^ The UCPC provides care to patients with CNCP but does not provide care to patients with known active illicit opioid use or to patients who are known to manipulate the formulation of their prescription opioid medications (e.g., snort or inject them) because patients meeting these criteria are referred to addiction medicine specialists separate from the UCPC. However, because there is overlap between CNCP and opioid dependence, some UCPC patients may meet criteria for mild-to-moderate OUD.

Most patients in this case series were referred to the UCPC by their primary prescriber due to inadequate pain control and/or opioid-related harms. At the time of referral to the UCPC, background patient information is collected and a clinical assessment is completed to obtain demographic information, diagnoses, medication history, pain history, psychiatric history, and substance use history. This information is stored within UCPC electronic medical records, which were retrospectively reviewed to obtain the information for this case series.

The pharmacists and physician at UCPC provided support to the patient’s primary prescriber to initiate bup/nal, including mentorship, education, and follow-up discussions, as needed. The case patients were identified for inclusion in this case series by retrospectively screening the electronic medical records of all UCPC patients to identify those whose treatment plan included a transition to bup/nal using a low-dose induction regimen for the treatment of CNCP.

**Figure 1.**
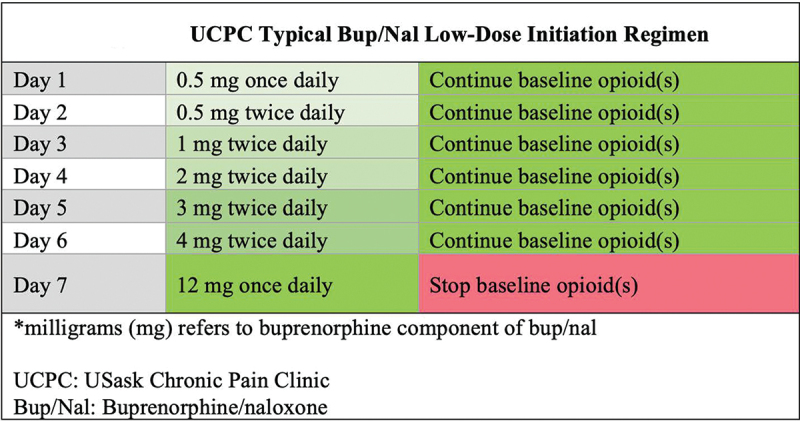
UCPC typical bup/nal low-dose initiation regimen. *Milligrams (mg) refers to buprenorphine component of bup/nal

Bup/nal initiation regimens were created using a standardized low-dose initiation protocol. The protocol involved gradual titration from 0.5 to 12 mg of sublingual bup/nal over a 7-day period while the patient continued to take their baseline opioid(s). The baseline opioid therapy was stopped on day 7 of the bup/nal initiation regimen. Although the low-dose initiation protocol was standardized, the regimens were adjusted by the UCPC clinicians based on patient-related factors such as baseline opioid use, comorbidities, and clinical status (Figure 1).^7,30^ During the low-dose initiation protocol, the UCPC offered flexibility in the discontinuation of baseline full opioid agonist based on patient preference (i.e., fear of withdrawal) and clinician assessment of their baseline dose of opioids. Patients were either tapered off their baseline opioids, stopped opioids in a stepwise approach (e.g., stopped hydromorphone immediate release on day 4 and then stopped hydromorphone long-acting on day 7), stopped baseline opioids abruptly without taper, or a combination of methods was used.

UCPC patient data were retrospectively collected from each patient’s electronic medical record. Extracted data points included patient demographics, clinical status pre and post bup/nal initiation (e.g., comorbidities, medications, baseline opioid use characteristics, pain rating scales). UCPC clinicians were encouraged to record a Clinical Global Impression–Improvement (CGI-I) score following each appointment with the patient. A CGI-I score is based on the clinician’s observation of the patient’s overall functioning compared to previous appointments and is a recommended monitoring parameter in CNCP treatment studies.^31^ Patient data were collected by one research assistant (D.K.) and reviewed and validated by a second research assistant (M.R.). K.H. provided supervisory support for data collection and resolved any data discrepancies. The extracted data were analyzed using descriptive statistics.

## Results

A total of 21 patients were initiated onto bup/nal for CNCP at the UCPC and were included in this case series. Patient baseline characteristics were identified as follows: the mean age was 53 years (range, 22–81), and the majority were female (66.7%). Patients had a median of 9 (range, 2–21) comorbidities. Several patients had comorbid psychiatric conditions, most commonly major depressive disorder (52.4%) and generalized anxiety disorder (23.8%). Additionally, 17 patients had a mild to severe score on the Patient Health Questionnaire-9 (PHQ-9; 81.0%) and 13 patients scored mild to severe on the General Anxiety Disorder-7 (GAD-7; 61.9%).^32,33^ The majority of patients did not have a formal substance use disorder (SUD) diagnosis (71.4%)—only 4 patients had a formal diagnosis of OUD (19.1%). Of the patients diagnosed with OUD met the following *Diagnostic and Statistical Manual of Mental Disorders*, fifth edition^34^ criteria: opioids are often taken in larger amounts than intended (*n* = 4); continued use despite knowledge of having a persistent or recurrent physical or psychological problem that is likely to have been caused or exacerbated by opioids (*n* = 4); a great deal of time is spent in activities necessary to obtain the opioid, use the opioid, or recover from its effects (*n* = 1); tolerance (*n* = 4); and withdrawal (*n* = 4). One of the patients had a remote history of illicit opioid use, but this was in remission at the time of referral and throughout care with the UCPC. Eight patients scored clinically significant (≥2) on the Prescription Opioid Misuse Index (POMI; 38.1%), 3 of whom were patients with a formal OUD diagnosis.

The most common pain diagnosis category was mixed/nociplastic pain (76.2%), followed by neuropathic pain (14.3%) and nociceptive pain (4.8%). The mean average numerical pain rating score at the initial visit was 7.2 out of 10 (range, 2–10), with a Brief Pain Inventory–Interference (BPI-I) score of 7.5 out of 10 (range, 0–10). Patients’ baseline pain was also assessed using the Central Sensitization Inventory (mean, 55.3; range, 28–87), Douleur Neuropathique en 4 Questions (mean, 3.8; range, 0–10), and Pain Catastrophizing Scale (mean, 28.1; range, 11–45).^35^

All patients were taking opioids for chronic pain at baseline, with hydromorphone being the most common opioid analgesic (61.9%). The mean daily oral morphine equivalent (OME) dose prior to bup/nal initiation was 197.8 mg (range, 22.5–960 mg). Patients were taking a median of 4 (range, 2–9) analgesics (opioid and nonopioid) and 11 (range, 6–25) total medications at baseline. Seventeen of the 21 patients were initiated onto bup/nal for CNCP (81.0%), and 4 were initiated for both CNCP and OUD (19.0%; Table 1).

**Table 1.** Baseline characteristics of 21 UCPC patients initiated onto bup/nal for CNCP.

Characteristics	Value (*n* = 21)
Demographic characteristics	
Sex, *n* (%)	
Female	14 (66.7)
Male	7 (33.3)
Age, years (range)	
Mean	53 (22–81)
Median	58
Clinical characteristics	
Total comorbidities, *n* (range)	
Mean	8.8 (2–21)
Comorbid mental disorders, *n* (%)	
Disclosed a comorbid mental health disorder	15 (71.4)
None disclosed	5 (23.8)
Not screened	1 (4.8)
PHQ-9 score at initial visit (0–27), *n* (%)^a^	
0–4 (none/minimal)	3 (14.3)
5–9 (mild)	4 (19.0)
10–19 (moderate to moderately severe)	6 (28.6)
20+ (severe)	7 (33.3)
Not screened	1 (4.8)
GAD-7 score at initial visit (0–21), *n* (%)^a^	
0–4 (no/low risk)	7 (33.3)
5–9 (mild)	4 (19.0)
10–14 (moderate)	3 (14.3)
15+ (severe)	6 (28.6)
Not screened	1 (4.8)
SUD diagnoses, *n* (%)	
OUD	4 (19.0)
Other SUD^b^	5 (23.8)
Mixed SUD features^c^	1 (4.8)
None	15 (71.4)
Opioid use characteristics	
Previous illicit opioid use, *n* (%)	1 (4.8)
Previous prescription opioid use, *n* (%)	21 (100)
Prescription opioids prior to initiation, *n* (%)	
Butorphanol	1 (4.8)
Fentanyl	3 (14.3)
Hydromorphone	13 (61.9)
Methadone	1 (4.8)
Morphine	3 (14.3)
Tramadol	2 (9.5)
Buprenorphine^d^	2 (9.5)
Unreliable OME conversion, *n* (%)^e^	6 (28.6)
Daily OME prior to initiation, mg (range)	*n* = 15
Mean	197.8 (22.5–960)
Median	90
Total analgesics prior to initiation, *n* (range)	
Median	4 (2–9)
Total medications prior to initiation, *n* (range)	
Median	11 (6–25)
POMI score at initial visit, *n* (%)^f^	
0	8 (38.1)
1	3 (14.3)
2+	8 (38.1)
Not screened	2 (9.5)
Indication for OAT	
CNCP	17 (81.0)
CNCP and OUD	4 (19.0)
Pain characteristics	
CNCP category, *n* (%)	
Mixed/nociplastic^g^	16 (76.2)
Neuropathic	3 (14.3)
Nociceptive	1 (4.8)
Unidentified	1 (4.8)
Average numerical pain rating scale at initial visit (1–10), *n* (range)	
Mean	7.2 (2–10)
BPI Interference score at initial visit (1–10), *n* (range)	*n* = 20
Mean	7.5 (0–10)
CSI score at initial visit (1–100), *n* (range)^h^	*n* = 19
Mean	55.3 (28–87)
DN4 score at initial visit (1–10), *n* (range)^i^	*n* = 20
Mean	3.8 (0–10)
PCS score at initial visit (1–52), *n* (range)^j^	*n* = 19
Mean	28.1 (11–45)

^a^PHQ-9 and GAD-7 scores are based on self-administered scales and are only used as screening tools at UCPC. Not considered diagnostic of major depressive disorder and GAD.

^b^Other SUD includes alcohol use disorder, mixed SUD.

^c^One patient met certain criteria for mixed SUD due to a history of crack cocaine use and prescription opioid misuse; however, the patient had no formal SUD diagnoses.

^d^Two patients were initiated onto transdermal buprenorphine prior to being initiated onto sublingual buprenorphine/naloxone due to insufficient pain control. Their opioids prior to initiation onto transdermal buprenorphine included morphine and tramadol, respectively.

^e^OME could not be calculated for six patients due to unreliable conversion. Opioids with unreliable OME conversion included butorphanol (*n* = 1, 10 mg/mL nasal spray, 5 sprays/day), sublingual fentanyl (*n* = 1, 75–125 μg SL daily), transdermal fentanyl (*n* = 2, mean: 31 μg/hour patch every 72 h), methadone (*n* = 1, 45 mg/day), and tramadol (*n* = 2, mean: 437.5 mg [425–450 mg]).

^f^POMI score ≥2 indicates a positive screen and greater probability of OUD.

^g^Mixed/nociplastic pain indicates elements of both nociceptive and neuropathic pain and/or central sensitization.

^h^A CSI score ≥30 indicates a positive screen and greater likelihood of central sensitization.

^i^A DN4 score ≥4 indicates a positive screen and greater likelihood of neuropathic pain.

^j^A PCS score ≥30 indicates a clinically relevant level of pain catastrophizing.

OAT = opioid agonist therapy, CSI = Central Sensitization Inventory, DN4 = Douleur Neuropathique en 4 Questions, PCS = Pain Catastrophizing Scale.

**Figure 2.**
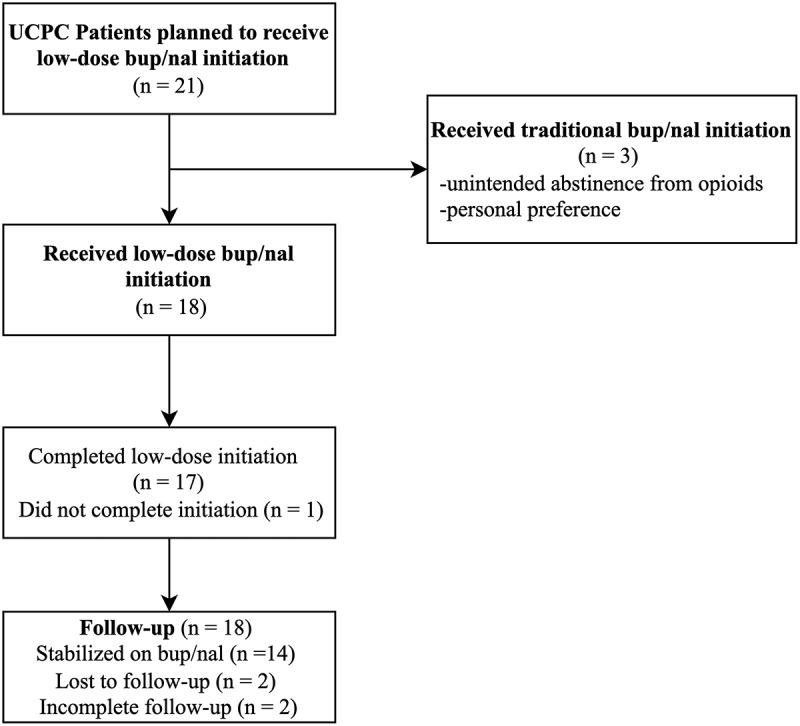
Flow diagram of UCPC patients’ buprenorphine/naloxone initiation.

All patients were planned to undergo low-dose initiation onto bup/nal; however, 3 of the 21 patients completed traditional bup/nal initiation regimens (14.3%). Traditional initiations occurred for the following reasons: 2 patients were initiated in acute care facilities following unintended abstinence from their baseline opioids, and 1 patient opted for a traditional initiation as a personal preference. These 3 patients were excluded, resulting in 18 patients who were included in the final data analysis (Figure 2).

**Figure 3.**
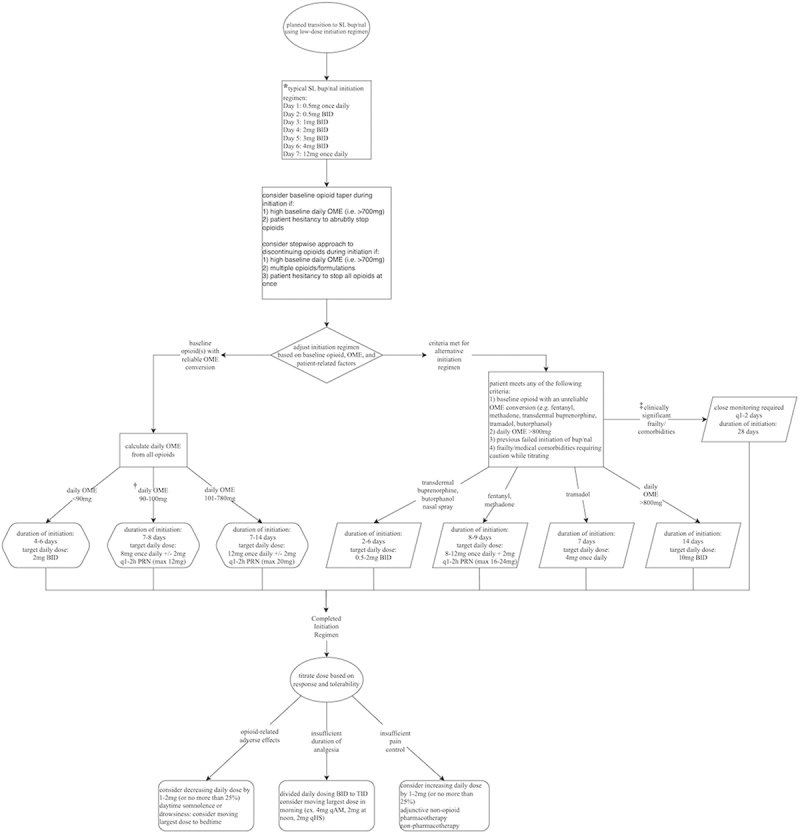
Low-dose buprenorphine/naloxone initiation regimens of UCPC patients.

Low-dose bup/nal initiation regimens varied based on baseline opioid(s), daily OME, and comorbidities/frailty (Figure 3). Of the 18 patients who underwent low-dose initiation to bup/nal, the median duration of initiation was 7 days (range, 2–28 days), and the median stop date of the previous opioid was on day 7 (range, 1–21 days). Two patients tapered off their baseline opioids (11.1%), 4 patients stopped their opioids in a stepwise approach (22.2%), and 13 patients opted to discontinue their opioids without tapering on the stop date (72.2%). Seventeen patients successfully completed the low-dose initiation regimen (94.4%; i.e., they were transitioned from their baseline full opioid agonist to bup/nal utilizing a low-dose initiation regimen) and the mean final daily bup/nal dose was 10.5 mg (range, 1–24 mg). One patient did not complete the initiation due to adverse effects, including nausea and drowsiness.

Baseline opioid: opioid used for CNCP prior to bup/nal initiation. Target dose: bup/nal dose at the end of the low-dose initiation regimen. Duration of initiation: the day of initiation at which all prior opioids were stopped AND target initial dose was reached.*Milligrams (mg) dosing refers solely to buprenorphine component of bup/nal.**†**Only two UCPC patients fit into the 90–100 mg daily OME category.‡Only one UCPC patient fit into the clinically significant frailty/comorbidities category. SL = sublingual; BID = twice daily; PRN = as needed.

During low-dose initiation, 12 patients experienced adverse effects (66.7%), with the most common being nausea (33.3%) and aches/pain (27.8%). Nine patients required supportive medications during the initiation (50.0%), with clonidine being the most common (38.9%). All adverse effects that occurred during the initiation of bup/nal resolved once the final maintenance dose of bup/nal was established.

Following low-dose initiation, 14 patients remained stable on bup/nal throughout their follow-up appointments with the UCPC (77.8%), 2 were lost to follow-up (11.1%), and 2 had not yet had follow-up appointments prior to data extraction (11.1%). The median CGI-I score after initiation was 2 (much improved) with a range between 1 (very much improved) and 5 (minimally worse). Patients were planned for follow-up at the UCPC until they reached a stable maintenance dose of bup/nal and could be managed by their primary outpatient health care team; thus, the duration of the follow-up period varied for each patient. At the final visit, patients were taking a median of 3 (range, 1–8) analgesics and 8.5 (range, 5–27) total medications, 25% and 22.7% reductions from baseline, respectively. Bup/nal doses were titrated, as needed, by the clinical team during the follow-up period to find the optimal dose for each individual patient. Patients were taking a mean daily bup/nal dose of 11.5 mg (range, 1–28 mg) at the final visit (Table 2).

**Table 2.** Results of 18 UCPC patients initiated onto bup/nal using a low-dose initiation regimen.

Characteristics	Value (*n* = 18)
Initiation characteristics	
Previous opioid stop date (day), *n* (range)^a^	
Mean	7.4 (1–21)
Median	7
Duration of initiation (days), *n* (range)^b^	
Mean	8.4 (2–28)
Median	7
Total daily bup/nal dose at the end of initiation, mg (range)^c^	
Mean	10.50 (1–24)
Initiation outcomes, *n* (%)^d^	
Successful	17 (94.4)
Unsuccessful	1 (5.6)
Psychosocial stressors present during initiation, *n* (%)^e^	
Yes	8 (44.4)
None disclosed	10 (55.6)
Adverse effects during initiation	
Symptoms, *n* (%)	
None	6 (33.3)
Anxiety	2 (11.1)
Irritability	2 (11.1)
Insomnia	2 (11.1)
Nausea	6 (33.3)
Gastrointestinal upset	2 (11.1)
Vomiting	3 (16.7)
Loss of appetite	1 (5.6)
Diarrhea	2 (11.1)
Aches/pain	5 (27.8)
Piloerection	1 (5.6)
Perspiration	2 (11.1)
Restlessness	2 (11.1)
Chills	1 (5.6)
Tremor	1 (5.6)
Supportive medications, *n* (%)^f^	
None	9 (50)
Acetaminophen	2 (11.1)
Clonidine	7 (38.9)
Hydroxyzine	1 (5.6)
Metoclopramide	1 (5.6)
Ondansetron	1 (5.6)
PEG3350	1 (5.6)
Patient status following completion of initiation, *n* (%)^g^	
Stable on bup/nal	14 (77.8)
Lost to follow-up	2 (11.1)
Incomplete follow-up	2 (11.1)
Pain improvement measures^h^	
Average numerical pain rating after initiation (1–10), *n* (range)	*n* = 5
Mean	4 (0–9)
BPI Interference score after initiation (1–10), *n* (range)	*n* = 2
Mean	5.9 (3.7–8.1)
CGI-I score after initiation (1–7), *n* (range)	*n* = 12
Mean	2.17 (1–5)
Median	2
Medication use characteristics	
Total analgesics at final visit, *n* (range)	*n* = 16
Median	3 (2–8)
Total medications at final visit, *n* (range)	*n* = 16
Median	8.5 (5–27)
Total daily bup/nal dose at final visit, mg (range)	*n* = 14
Mean	11.5 (1–28)

^a^Opioid stop date is defined as the day of initiation at which all prior prescription opioids were stopped.

^b^Duration of initiation is defined as the day of initiation at which all prior prescription opioids were stopped and target initial dose was reached.

^c^Total daily dose at the end of initiation is defined as the maximum allowable total daily dose on the final day of initiation.

^d^Successful initiation is defined as completion of the bup/nal initiation regimen and abstinence from previous opioids.

^e^Psychosocial stressors are defined as any situations/circumstances that create an unusual or intense level of stress in the patient’s life (excluding chronic pain).

^f^Supportive medications are defined as any medications used to relieve symptoms of withdrawal during initiation.

^g^Patient status following initiation was monitored by the UCPC care team. Patient follow-ups were planned to be conducted until the patient was on a stable maintenance dose of bup/nal and could be managed by their personal health care team. Two patients were lost to follow-up prior to establishing a maintenance dose. Two patients did not complete follow-up prior to the case series data collection.

^h^Pain improvement measures were collected at follow-up visits after completion of initiation. There were no standardized time frames during which these values were collected.

## Discussion

This article represents the largest published case series of patients who were successfully transitioned to sublingual bup/nal using a low-dose initiation method for the treatment of CNCP. The low-dose initiation regimens that are reported in this study were generally well tolerated by patients, with only one failed initiation and no serious complications (i.e., no opioid toxicity events or death). The most common adverse effect of nausea may have been due to precipitated opioid withdrawal or due to buprenorphine itself, which carries a risk of nausea of 16.1%.^11^ This study thus adds to the available data to support the use of low-dose bup/nal initiation regimens in the setting of CNCP and LTOT.

At present, there is limited published literature on bup/nal initiation strategies for the purpose of CNCP. Published literature on patients transitioned to bup/nal using a low-dose initiation method consists only of case series and case reports. The largest previously published case series of patients being transitioned from full opioid agonists to bup/nal for CNCP using a low-dose initiation protocol consisted of eight patients.^8^ The eight patients were all experiencing opioid dependence and two met *Diagnostic and Statistical Manual of Mental Disorders*, fifth edition criteria for OUD. Similar to the patients in our case series and the other published cases, Robbins et al.^8^ found that low-dose initiation regimens were generally well tolerated, and the majority of patients experienced improved pain control following the transition. Our case series adds to the growing evidence that low-dose initiation of bup/nal is a feasible treatment option for patients experiencing adverse effects, uncontrolled pain, or opioid misuse while taking opioids for CNCP.

Limitations of this study include practitioner bias, inconsistencies in collected data at appointments, and the absence of reported withdrawal and pain scales once patients started their bup/nal initiation regimens. The variability in practitioner reporting of clinical response may lead to unreliable data. It is not possible to distinguish adverse effects reported at appointments as opioid withdrawal, precipitated withdrawal, or adverse effects of bup/nal, and therefore it is not possible to determine that low-dose initiation strategies used by UCPC avoid opioid withdrawal. Clinical Opiate Withdrawal Scale (COWS) scores and pain scales were not routinely completed during follow-up appointments for a variety of reasons, including time limitations, negative impact of measurement-based care tools on clinician patient interactions, and logistical challenges of appointments (i.e., many were completed virtually).^36^ Given the complexity of CNCP conditions, pain scales completed at one point in time may not accurately reflect a patient’s progress.

The generalizability of this case series is limited by the cohort’s small sample size and retrospective data collection. Additionally, the cohort in this study exhibited higher rates of opioid misuse (38.1%) and OUD (19.1%) than published averages, which indicates that the rate of misuse in patients taking opioid therapy for CNCP is 21% to 29% and the rate of addiction is 8% to 12%.^37^ However, the rates of opioid misuse and OUD in patients with CNCP are not well established in published literature. Moreover, the elevated rates observed in this study could be attributed to selection bias, because the patients in this case series were referred to the UCPC based on their complexity and were chosen to transition to bup/nal based on the UCPC clinician’s experience in caring for patients with CNCP and may not be reflective of all patients with CNCP. However, heterogeneity of the patients presented in this cohort in terms of demographics, comorbidities, opioid use, and pain characteristics highlights the adaptability of low-dose bup/nal initiation regimens.

The UCPC staff is committed to providing person-centered care, which includes tailoring pharmacotherapy to the patient’s needs, as is demonstrated in this case series, which highlights the importance of flexible initiation regimens. Given that traditional bup/nal initiation regimens require patients to be in mild-to-moderate withdrawal prior to initiation, these patients would have been higher risk for worsening pain and withdrawal symptoms had they undergone a traditional induction protocol.^13,15^ The initiation regimens used in this study led to an improvement in global functioning and pain scores, further supporting the feasibility of the protocol.

Further research is needed to describe the relationship between CNCP and OUD. We have noted that many patients with CNCP may have a mild OUD that is not formally diagnosed. Moreover, randomized controlled trials evaluating the effectiveness of low-dose initiations versus traditional initiations for reducing psychiatric and physical stress associated with opioid withdrawal during bup/nal initiation are required to support low-dose initiation regimens as the standard of care.

## Conclusion

This article represents the largest case series of patients who were successfully transitioned to sublingual bup/nal using a low-dose initiation method for the treatment of CNCP. The initiation regimens were generally well tolerated, flexible, and tailored to meet the patient’s needs and led to improved global functioning. A low-dose initiation regimen of bup/nal may be considered as an induction method for patients transitioning to bup/nal for the treatment of CNCP, although the results should be interpreted cautiously based on the small sample size.

## Appendix 1. Summary of published literature of adult patients transitioned to bup/nal for CNCP using a low-dose initiation regimen.

**Table:**

Study	Study design	Patient characteristics	Intervention	Outcomes
Kornfeld and Reetz^21^	Case series	Three opioid-dependent patients with CNCP taking short-acting opioid medications (daily OME range, 40–142.5 mg)^a^	Transition from full opioid agonist to bup/nal (0.75–32 mg bup/day) using low-dose buprenorphine transdermal patch as a bridge	Successful transition to bup/nal for all patients without precipitated withdrawal or significant adverse effects
Sandhu et al.^22^	Case report	59-year-old female with acute on chronic pain and history of OUD (daily OME approximately 95 mg)	Transition from oxycodone to bup/nal (12 mg once daily) using a low-dose initiation regimen in a postoperative inpatient setting	Successful transition to bup/nal without a period of withdrawal and reported full relief of head and neck pain
Robbins et al.^8^	Case series	Eight patients age 53 to 70 years with CNCP and opioid dependence (daily OME range, 75–240 mg)^b^	Transition from full opioid agonist to bup/nal (16–24 mg bup/day in two to four divided doses) using a low-dose protocol in an outpatient general medicine practice	Six patients remained on bup/nal and reported improved analgesia
Becker et al.^23^	Case series	Six patients with chronic pain taking high-dose long-term opioids (daily OME range, 105–390 mg)	Transition from full opioid agonist to bup/nal (6–16 mg/day in three to four divided doses) using a standardized low-dose initiation protocol in an outpatient multidisciplinary pain clinic	Successful transition to bup/nal without opioid withdrawal symptoms for all patients. One patient resumed full opioid agonist therapy due to inadequate pain control
Crum et al.^24^	Case report	76-year-old female with CNCP and an intrathecal hydromorphone-bupivacaine pump (daily OME inconsistent)	Transition from intrathecal hydromorphone-bupivicaine to bup/nal (8 mg bup/day divided BID) using a low-dose initiation regimen in an inpatient hospital setting	Successful transition to bup/nal while providing adequate pain control and preventing withdrawal and ventilatory depression. Patient self-discontinued bup/nal without use of other opioids
Lee et al.^25^	Case report	66-year-old female with inadequately controlled postoperative pain	Transition from oxycodone and methadone to bup/nal (12 mg bup/day) using a rapid low-dose initiation regimen in an inpatient postoperative setting	Successful transition to bup/nal with no significant withdrawal symptoms and improved pain, function, and mood
Tara et al.^26^	Case report	27-year-old female unable to tolerate PO intake with uncontrolled acute on chronic pain taking opioid analgesics	Transition from full opioid agonist to bup/nal (8 mg bup/day) using a low-dose initiation regimen in an inpatient hospital setting	Successful transition to bup/nal with improved pain relief. Patient elected to taper off bup/nal prior to discharge
Leyde et al.^19^	Case report	22-year-old female with sickle cell disease and CNCP taking oxycodone (daily OME 960 mg)	Transition from oxycodone to bup/nal (40 mg bup/day in four divided doses) using low-dose buprenorphine transdermal patch as a bridge in an inpatient hospital setting	Successful transition to bup/nal with minimal withdrawal symptoms and improved functioning
MacAusland-Berg et al.^27,c^	Case report	80-year-old female with inadequately controlled chronic pain	Transition from intranasal butorphanol to bup/nal (4 mg bup/day divided BID) using a low-dose initiation regimen in an outpatient interdisciplinary chronic pain clinic	Successful transition to bup/nal with minimal withdrawal symptoms and no pain exacerbations

^a^Excludes OME calculation of one patient due to unreliable conversion.

^b^Contains an unreliable OME conversion of 100ug fentanyl to 240 mg OME.

^c^Case report details a UCPC patient that was included in presented case series.

BID = twice daily; PO = per oral.
